# Supplementary material for: Monocyte-Derived miRNA-1914-5p Attenuates IL-1β–Induced Monocyte Adhesion and Transmigration
Source: Int J Mol Sci. 2023 Feb 1;24(3):2829. doi: 10.3390/ijms24032829 (PMC9917334; doi:10.3390/ijms24032829)
Supplement: Supplementary file 1 [file ijms-24-02829-s001.zip › ijms-1767312-supplementary.pdf]

Supplementary material

Monocyte-derived miRNA-1914-5p attenuates IL-1β–induced monocyte adhesion and transmigration

Kohki Toriuchi, Toshie Kihara, Hiromasa Aoki, Hiroki Kakita, Satoru Takeshita, Hiroko Ueda, Yasumichi Inoue, Hidetoshi Hayashi, Yohei Shimono, Yasumasa Yamada, and Mineyoshi Aoyama

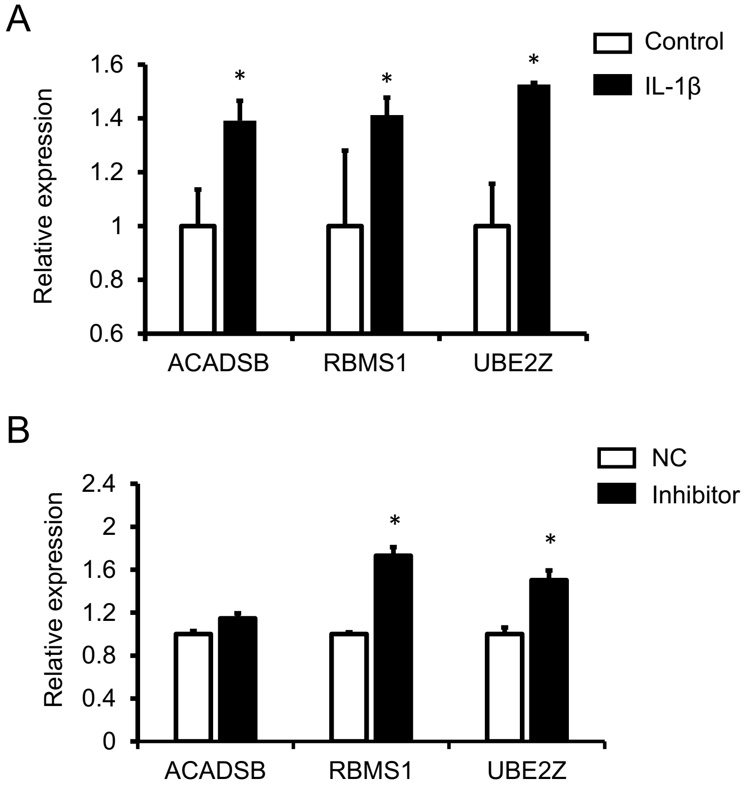

Figure S1. Expression of miR-1914–5p target genes.
(A) miR-1914–5p target gene expression was examined by real-time RT-PCR. THP-1 cells were incubated with or without IL-1β for 24 h. (B) THP-1 cells transfected with an miR-1914–5p or negative control (NC) inhibitor. Data are the mean ± SEM (n=3 in each group). *p<0.05 compared with the control group (A) or NC-transfected group (B).

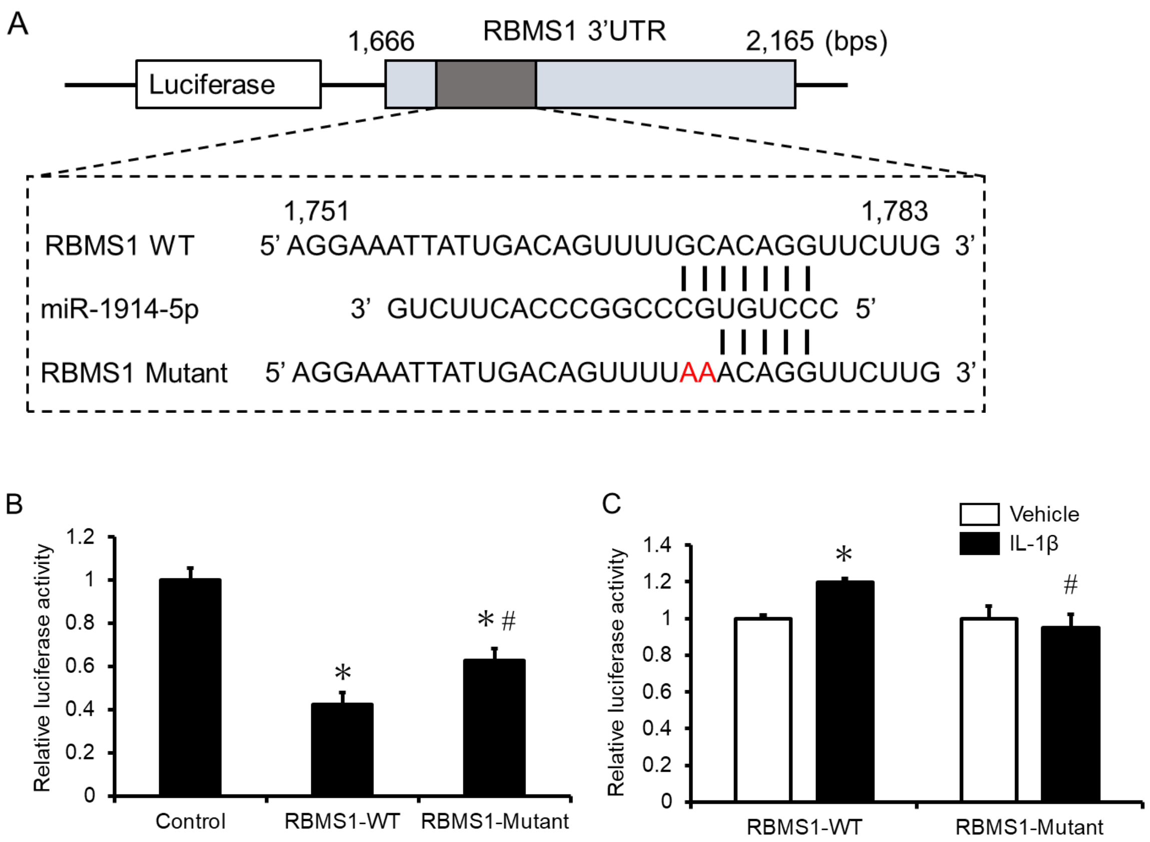

Figure S2. MicroRNA miR-1914–5p directly targets the 3′UTR region of the RBMS1 mRNA.
(A) Schematic representation of the predicted miRNA-1914-5p target recognition sequences within the 3′-UTR of the RBMS1 mRNA, and of the mutant in which two nucleotides within the putative miR-1914-5p target sequence were mutated. Numbers correspond to nucleotide positions in RBMS1 sequence (GenBank: NM_ 016836.4). WT: wild-type.
(B) Luciferase activity of pGL3 constructs encoding the WT or mutant version of the RBMS1 3′-UTR in THP-1 cells. Luciferase activities were measured after 24 h and normalized by Renilla luciferase activities. Data are presented as the mean ± SEM (n=6 in each group). *p<0.05 compared with the pGL3-MC control vector. #p<0.05 compared with the RBMS1-WT 3′-UTR.
(C) Relative luciferase activities of RBMS1-WT 3′UTR and RBMS1-Mutant 3′UTR in THP-1 cells treated with vehicle or IL-1β. Luciferase activities were measured after 24 h and normalized by Renilla luciferase activities. Data are presented as the mean ± SEM (n=3 in each group). *p<0.05 compared with the RBMS1-WT under vehicle treatment. #p<0.05 compared with the RBMS1-WT under IL-1β treatment.
